# NME1 Protects Against Neurotoxin-, α-Synuclein- and LRRK2-Induced Neurite Degeneration in Cell Models of Parkinson’s Disease

**DOI:** 10.1007/s12035-021-02569-6

**Published:** 2021-10-08

**Authors:** Jayanth Anantha, Susan R. Goulding, Eszter Tuboly, Adam G. O’Mahony, Gerard M. Moloney, Gareth Lomansey, Cathal M. McCarthy, Louise M. Collins, Aideen M. Sullivan, Gerard W. O’Keeffe

**Affiliations:** 1 Department of Anatomy and Neuroscience, University College Cork, Cork, Ireland; 2 Department of Physiology, University College Cork, Cork, Ireland; 3 Department of Pharmacology and Therapeutics, University College Cork, Cork, Ireland; 4 APC Microbiome Ireland, University College Cork, Cork, Ireland; 5 Parkinson’s Disease Research Cluster (PDRC), University College Cork, Cork, Ireland

**Keywords:** Parkinson’s disease, Neuroprotection, Dopaminergic, Axon, Alpha-synuclein Mitochondria

## Abstract

Parkinson’s disease (PD) is a neurodegenerative disease characterised by the progressive degeneration of midbrain dopaminergic neurons, coupled with the intracellular accumulation of α-synuclein. Axonal degeneration is a central part of the pathology of PD. While the majority of PD cases are sporadic, some are genetic; the G2019S mutation in leucine-rich repeat kinase 2 (LRRK2) is the most common genetic form. The application of neurotrophic factors to protect dopaminergic neurons is a proposed experimental therapy. One such neurotrophic factor is growth differentiation factor (GDF)5. GDF5 is a dopaminergic neurotrophic factor that has been shown to upregulate the expression of a protein called nucleoside diphosphate kinase A (NME1). However, whether NME1 is neuroprotective in cell models of axonal degeneration of relevance to PD is unknown. Here we show that treatment with NME1 can promote neurite growth in SH-SY5Y cells, and in cultured dopaminergic neurons treated with the neurotoxin 6-hydroxydopamine (6-OHDA). Similar effects of NME1 were found in SH-SY5Y cells and dopaminergic neurons overexpressing human wild-type α-synuclein, and in stable SH-SY5Y cell lines carrying the G2019S LRRK2 mutation. We found that the effects of NME1 require the RORα/ROR2 receptors. Furthermore, increased NF-κB-dependent transcription was partially required for the neurite growth-promoting effects of NME1. Finally, a combined bioinformatics and biochemical analysis of the mitochondrial oxygen consumption rate revealed that NME1 enhanced mitochondrial function, which is known to be impaired in PD. These data show that recombinant NME1 is worthy of further study as a potential therapeutic agent for axonal protection in PD*.*

## Introduction

Parkinson’s disease (PD) is the most common age-related motoric neurodegenerative disease [1]. It affects 1% of the population over the age of 60 years [2, 3]. Loss of striatal dopamine is recognised as the underlying pathophysiological cause of motor dysfunction [4–6]. This results from the progressive degeneration of midbrain dopaminergic neurons in the substantia nigra (SN) and their axons, which project to the striatum as the nigrostriatal pathway [4–6]. PD is also characterised by the accumulation of intracellular α-synuclein aggregates, called Lewy bodies and Lewy neurites, in neuronal soma and neurites, respectively [7, 8]. While the majority of cases of PD are sporadic, there is a significant genetic component to some cases. Mutations in *SNCA* [9, 10] and *LRRK2* [11] are arguably the most well-known mutations that are responsible for autosomal dominant PD. The glycerine to serine substitution (G2019S) is the most common mutation in *LRRK2* and is found in 1% of sporadic cases, as well as in 4% of patients with a genetic form of PD [12]. Irrespective of the cause, dopamine replacement strategies can, for a time, manage the symptoms of PD [13], but there is a critical need for disease-modifying therapies [14]. One therapeutic approach that is the subject of an intensive research effort is neurotrophic factor therapy, which is the application of neurotrophic factors to stop dopaminergic neurodegeneration.

One such neurotrophic factor is growth differentiation factor 5 (GDF5), a member of the transforming growth factor (TGF)-β superfamily [15–17]. GDF5 has been shown to have neuroprotective effects in rat models of PD, specifically in 6-hydroxydopamine (6-OHDA) lesion models [18, 19], and more recently in the AAV-α-synuclein model [14]. Although GDF5 is known to signal through the canonical BMP-Smad pathway (for review see [20]), the downstream mediators of its mechanism of action are largely unknown. We have recently shown that the beneficial effects of GDF5 on neurite growth are mediated by nucleoside diphosphate kinase A, encoded by the *NME1* gene [21]. This is important as neurite degeneration is considered to be an early part of the pathology of PD (for reviews see [22, 23]).

NME1 is expressed in multiple regions of the developing and adult mouse brain, including the midbrain [21, 24]. d NME1 overexpression in oligodendrocyte progenitors induces the acquisition of a neuronal fate [25]. Our recent study showed that GDF5 treatment of SH-SY5Y cells resulted in the upregulation of NME1, and that overexpression of NME1 was necessary and sufficient for basal and GDF5-induced neurite outgrowth [21]. NME1 has been reported to be present in the extracellular environment of the central nervous system (CNS) and as a multimer in the extracellular environment of stem cells, promoting differentiation [26]. In agreement with this, we reported that exogenous application of recombinant NME1 promoted neurite growth of SH-SY5Y cells, and also of dopaminergic neurons in primary cultures of embryonic day (E)14 rat ventral mesencephalon (VM) [21]. It has also been reported that extracellular NME1 is able to stimulate neurite outgrowth in explants of embryonic and adult dorsal root ganglia in vitro [27]. Other studies have shown that NME1 is secreted from E14 mouse cortical neurons in an *in vitro* model of traumatic brain injury [28]. However, despite these findings, it is not yet clear whether extracellular NME1 can protect neurons from injury. To address this question, we studied the effects of recombinant NME1 on neurite growth in cell models of PD.

## Materials and Methods

### Cell Culture

Human SH-SY5Y cells (ATCC; CRL-2266) were cultured in Dulbecco’s Modified Eagle’s Medium (DMEM) (Sigma D5796) supplemented with 10% foetal bovine serum (FBS), 1% sodium pyruvate, 1% l-glutamate, 1% penicillin–streptomycin and 1% non-essential amino acids (all from Sigma) and maintained at 37 °C with 5% CO_2_. To generate primary cultures of dopaminergic neurons, the VM was dissected from E14 rat embryos following their removal by laparotomy from time-mated Sprague Dawley rats culled by terminal anaesthesia and decapitation, under full ethical approval. VM tissue was collected in Hanks balanced salt solution (HBSS). The tissue pieces were then resuspended in 0.1% trypsin HBSS and incubated at 37 °C for 5 min at 5% CO_2_. Trypsin was inactivated by the addition of FBS followed by centrifugation at 1100 rpm for 5 min at 4 °C. The pellet was resuspended in 1 ml of complete media (DMEM/F12, 33 mM d-glucose, 1% l-glutamine, 1% FBS, supplemented with 2% B27) and triturated using a sterile glass pipette. The cells were plated at a density of 1 × 10^5^ cells per well in 0.5 ml media in a poly-d-lysine-coated 24-well plate.

### Plasmid and siRNA Transfection of SH-SY5Y Cells

SH-SY5Y cells were transfected using TransITX2® reagent (Mirus Bio, Cat # 6000) as per the manufacturer’s guidelines. 500 ng of plasmid or 10 nM of siRNA was mixed with 1.5 µl of TransITX2® in 50 µl of antibiotic-free and FBS-free Minimum Essential Media (MEM; Sigma) and incubated for 20 min at room temperature, before the mixture was added to the cells. Where indicated, cells were transfected with the following mammalian expression plasmids or siRNAs: pEF-DEST51 coding for wild-type LRRK2 (WT LRRK2) pDEST51-LRRK2-WT (Addgene #25080; http://n2t.net/addgene:25080; RRID: Addgene_25080) [29] or G2019S LRRK2 (Addgene #29401; http://n2t.net/addgene:29401; RRID:Addgene_29401) were gifts from Dr. Mark Cookson. Transgene expression was regulated by an EF1α/T7 promoter upstream of LRRK2 (WT or G2019S) coding region, and a blasticidin resistance gene downstream of an EM7 promoter. Wild-type α-synuclein (WT SNCA) and pEGFP-C1 plasmids were a gift from Dr. David Rubinsztein (Addgene # 40822; http://n2t.net/addgene:40822; RRID:Addgene_40822) [30]. Where indicated, SH-SY5Y cells were transfected with a GFP expression plasmid with 10 nM of one of the following siRNAs: Scrambled (siSCR) (F:5′-CGU UAA UCG CGU AUA AUA CGC GUAT-3′ and R: 5′AUA CGC GUA UUA UAC GCG AUU AAC GAC-3′), or siRNAs targeting ROR2 (siROR2) (F: 5′-UCU GAA AGG UUA CUU UCU GAA UUT T-3′ and R: 5′-AAA AUU CAG AAA GUA ACC UUU CAG AGU-3′) or RORα (siRORα) F:5′-AUU GCU UUU ACG GUA AAC CAA GAT A-3′ and R: 5′-UAU CUU GGU UUA CCG UAA AAG CAA UGU-3’). The duplex siRNA was purchased from IDT.

### Viral Transduction of Dopaminergic Neurons in E14 Rat VM Cultures

Adeno-associated viral vectors (AAV) 2/6 were generated by Vector Biosystems Inc (Philadelphia, USA), with a synapsin‐1 promoter driving the expression of human wild‐type α-synuclein or GFP [kind gifts from Dr Eilis Dowd (National University of Ireland Galway) and Prof. Deniz Kirik (Lund University)]. The final viral titres for AAV2/6-α-Synuclein (AAV-αSyn) and AAV2/6-GFP (AAV-GFP) were 5.2 × 10^13^ gc/ml and 5.0 × 10^13^ gc/ml, respectively. For transduction experiments, 1.5 × 10^4^ cells prepared from E14 rat VM were plated per well in a 96-well plate and transduced with either AAV-αSyn or AAV-GFP to achieve a multiplicity of infection (MOI) of 2.0 × 10^5^. Cells were then cultured with or without 100 ng/mL NME1 in two experimental paradigms: (1) NME1 treatment beginning at the time of infection (combined treatment), or (2) NME1 treatment starting 5 days after viral infection (delayed treatment). NME1 was added daily and the end-point was 10 days in both experiments. At this end-point, the cultures were fixed and processed for tyrosine hydroxylase (TH) immunocytochemistry. The lengths of individual axons of at least 30 TH-positive neurons were analysed per experiment using Image J. Each experiment was repeated three times.

### Establishment and Cultivation of Cell Lines Stably Expressing Wild-Type or G2019S LRRK2

SH-SY5Y cells were plated at 3 × 10^4^ cells per well of a 6-well plate and were transfected with WT LRRK2 or G2019S LRRK2 at 36 h post-transfection. The media was replaced with culture media containing 10 µg/mL of blasticidin and the cells were then cultured for a further 72 h. All dead and floating cells were removed with a media change and subsequently cultured in fresh media containing 10 µg/mL of blasticidin until they were 30–40% confluent. Cultures were then trypsinised and serially diluted in 96-well plates to ensure that only a single cell was present in each well, to establish a homogenous clonal population. A single homogenous clonal population was established for each of the LRRK2 constructs (i.e. WT and G2019S), the cells were expanded, and stocks were stored in the vapour phase of liquid nitrogen. The cells were expanded and cultured in culture media consisting of DMEM supplemented with 10% FBS, 1% sodium pyruvate, 1% l-glutamate, 1% penicillin streptomycin and 1% non-essential amino acids with the addition of 5 µg/ml of blasticidin to ensure selective pressure and stable expression of the constructs. Cells were plated and maintained in blasticidin-free media in 12-well or 24-well cell culture plates for NME1 treatment. The cells were cultured for up to 10 passages once revived from liquid nitrogen. The expression of the LRRK2 (WT and G2019S) proteins in the stably-transfected SH-SY5Y cells was confirmed by immunostaining for LRRK2 and for the His-tag on the expressed LRRK2 proteins.

### Western Blotting and Densitometry

Parent, WT LRRK2 and G2019S LRRK2 cell lines were plated at a density of 2 × 10^6^ cells in a 6-well plate and allowed to grow for 72 h before being washed twice with sterile PBS and then lysed in RIPA buffer supplemented with a cocktail of protease inhibitors (Roche molecular Biochemicals), 1 mM sodium fluoride and 1 mM sodium orthovanadate for 20–30 min on ice. The lysates were then centrifuged at 13,200 RPM for 20 min and the resultant supernatants were tested for protein concentrations using the BCA method (Pierce: Cat no. 23227). The samples were then mixed with 1 × sample loading buffer containing (5xSLB: 70 ml glycerol, 30 ml ddH2O, 2.5 g of SDS, 0.606 g of tris base with 5–6% β-mercaptoethanol) and were resolved on a 10% SDS-PAGE gel. The proteins were transferred onto a PVDF membrane which was then blocked in a blocking buffer containing 5% BSA in 10 mM PBS/TBS with 0.1% Tween20 for 2 h at room temperature. The membranes were washed three times with washing buffer containing 10 mM PBS with 0.1% Tween20. Following this, the membranes were incubated in either anti-GAPDH (SCBT: SC-47724, 1:1000) or anti-LRRK2 (ab133474, 1:1000) and incubated for 12–16 h at 4 °C. Following this, the blots were washed three times with washing buffer. The blots were then labelled with secondary antibodies in blocking buffer using the following HRP-conjugated secondary antibodies: anti-mouse 1:10,000 (ThermoFisher: Cat No. A27025) or anti-rabbit 1:2000 (ThermoFisher: Cat No. 31460). Following three washes in washing buffer, the blots were developed using ECL kit (Thermo Scientific: Cat No. 32106) and the luminescence was captured on Laser 3000 luminescent image analyser Fujifilm, with the duration of exposure optimised for each protein. These images were used to perform densitometric analysis using  ImageJ (Version 1.53a).

### Immunocytochemistry

Where indicated, cells were fixed in cold 4% paraformaldehyde for 10–15 min. The cells were washed three times in 10 mM PBS and then permeabilised in 10 mM PBS and 0.2% Triton-X100 (PBS-Tx) for 30 min at room temperature. Following a 5-min wash in 10 mM PBS, non-specific binding was blocked by incubation in a blocking buffer (1% bovine serum albumin (BSA) solution in PBS-Tx) for 1 h at room temperature. The cells were then washed for 3 × 5 min in PBS-Tx, before being incubated in primary antibody diluted in blocking buffer for 12–16 h at 4 °C. The following antibodies were used: anti-NME1/NDKA (1:200, CST #3345), anti-LRRK2 (1;500, Abcam ab133474), anti-TH (1:500, Millipore AB152) anti-His (1:500, Invitrogen MA1-21315). Following removal of the primary antibody, cells were washed for 3 × 5 min in PBS-Tx, and then incubated with either Alexa Fluor® 594 anti-rabbit (Cat No. A11012), Alexa Fluor® 594 anti-mouse (Cat No. A11005), Alexa Fluor® anti-rabbit 488 (Cat No. A21206) for 2 h at room temperature diluted at 1:500. The secondary antibody was removed and the cells were washed for 3 × 5 min in PBS-Tx, counterstained with DAPI and imaged using an Olympus IX 71 inverted microscope.

### Recombinant NME1 and BAY 11-7085 (NF-κB Inhibitor) Treatment and Neurite Length Analysis

Cells were treated with recombinant human NME1 (Novus Bio, Cat No. NBP2-52250) at a concentration 100 ng/mL for the indicated duration, based on our previous work [21]. SH-SY5Y cells and primary cultures of E14 rat VM were treated with 5 µM of 6-OHDA, along with either 50 ng/ml (2.5 nM), 100 ng/ml (5 nM), 150 ng/ml (7.5 nM), or 200 ng/ml (10 nM) of recombinant NME1, as indicated. Three wells were used per treatment group. Cells were treated at 24 h and at 48 h. In transfection experiments, SH-SY5Y cells were transfected with 500 ng of GFP or eGFP-WT-SNCA and cultured with or without NME1 for 48 h. The LRRK2 WT and G2019S mutant cell lines were plated at 2 × 10^4^ cell per well in a 24-well plate and cultured with or without of NME1 for 48 h. For the siRNA experiments, SH-SY5Y cells were co-transfected with GFP and siSCR, siROR2 or siRORα and cultured with or without NME1 for 48 h. In experiments involving the application of Bay 11-0785 (an inhibitor of NF-κB hereafter referred to as Bay; Sigma Cat No. B5681), SH-SY5Y cells were treated with 2 µM of Bay and 100 ng/ml of NME1 and cultured for 48 h. Phase contrast images or fluorescent images of GFP- or TH-positive cells were captured using Olympus IX 71 inverted microscope at X20 magnification. The neurite lengths of five cells per field from five images per well were measured using Image J and the averages from each independent experiment were used to compare the percentage change in neurite length between groups. Each experiment was repeated at least three times.

### RNA Extraction, cDNA Synthesis and qRT-PCR

For PCR experiments, SH-SY5Y cells were plated in a 12-well plate at a density of 5 × 10^5^ cells per well. Cells were transfected at 24 h after plating with a GFP reporter plasmid, along with either a scrambled control siSCR, siRORα, or siROR2 siRNA. RNA was extracted at 48 h post-transfection, using RNeasy Plus kit (Qiagen) in accordance to the manufacturer’s instructions. RNA concentration was quantified using the ND-1000 spectrophotometer (Nanodrop). Following RNA extraction, equal amounts of RNA were reverse-transcribed to cDNA using a high-capacity cDNA reverse transcription kit (Applied Biosystems, Life Technologies, Carlsbad, CA). All cDNA was stored at − 80 °C until time of assay. Gene expression was analysed using probe-based assays and gene-specific primers on an LC480 Lightcycler II (Roche Scientific). Expression levels were calculated as the average of three technical replicates for each biological sample from all three groups, relative to β-actin expression. Fold changes were calculated using the ΔΔCt method [31]. PCR primers were designed using published sequence data obtained from the NCBI database. All PCR probes were specific PrimeTime® qPCR assays: Human Actinβ ACTB (Hs.PT.39a.22214847), Human ROR2 (Hs.PT.58.22908006) and Human RORA (Hs.PT.58.27208579).

### NF-κB Luciferase Gene Reporter Assay

SH-SY5Y cells were plated at a density of 1 × 10^5^ for 12 h in a 24-well plate and transfected with 100 ng of NF-κB firefly luciferase reporter plasmid (a kind gift from Prof. Justin McCarthy, UCC [32]), 1 ng of renilla vector and a GFP-expressing plasmid. The total amount of plasmid DNA was made up to 500 ng by the addition of an empty vector. At 12 h post-transfection, the transfected cells were treated with or without 100 ng/ml recombinant NME1 and incubated at the aforementioned conditions for 16 h. The cells were then washed with 10 mM sterile PBS and lysed in passive lysis buffer (Promega, Cat no. E1941) and the samples were then snap-frozen to improve cell lysis. The samples were subsequently centrifuged at 13,200 RPM for 30 min at 4 °C. The lysates were then analysed at room temperature on a Veritas luminometer. Firefly luciferase activity was normalised to renilla luciferase reporter activity before further statistical analysis.

### Seahorse Assay to Assess Mitochondrial Function

SH-SY5Y cells were plated in a V3-PS TC-treated Agilent Seahorse XF96 cell culture plate at a density of 3 × 10^4^ cells per well. It was ensured that SH-SY5Y cells were at a passage number lower than 30 for each run. At 24 h after plating, the cells were treated with 100 ng/ml of NME1 for a duration of 48 h. The oxygen consumption rate (OCR) was measured using a Cell Mito Stress Test Kit (Cat No. 103015-100) from Agilent according to the manufacturer’s instructions using a MitoXF96 analyzer. The kit focuses on several aspects of cellular respiration using oligomycin (ATP-synthase inhibitor), FCCP (Protonophore, uncouples mitochondrial oxidative phosphorylation) and rotenone (inhibits mitochondrial complex I, thereby inhibiting mitochondrial electron transport chain). The OCR values were used to calculate basal respiration, proton leak, maximal respiration and ATP production rate. The cells were lysed in RIPA buffer after the conclusion of the assay and the protein levels were measured using bicinchoninic acid (BCA) assay ThermoFisher Scientific (Cat No. 23227) to ensure uniform protein content across groups.

### Gene Co-expression and Gene Ontology Analysis

A list of all genes that were significantly co-expressed with NME1 in the cerebral cortex (*n* = 130) and SN (*n* = 101) was generated by performing a pair-wise Pearson’s correlation analysis with Bonferroni’s testing for multiple correction using gene expression data from GSE:60863 and using the genomic analysis and visualization platform (https://hgserver1.amc.nl/cgi-bin/r2/main.cgi). Gene ontology (GO) enrichment analysis was performed using the gene ontology platform (www.geneontology.org).

### Statistical Analysis

Statistical analysis was performed using GraphPad Prism version 8 (©2018 GraphPad software, CA USA). Two-way or one-way ANOVAs were performed with appropriate *post-hoc* tests as indicated in the figure legends. Student’s *t*-tests were performed wherever applicable. Each experiment was independently performed at least three times.

## Results

### Recombinant NME1 Protects Against the Detrimental Effects of 6-OHDA on Neurite Growth in SH-SY5Y Cells

**Fig. 1:**
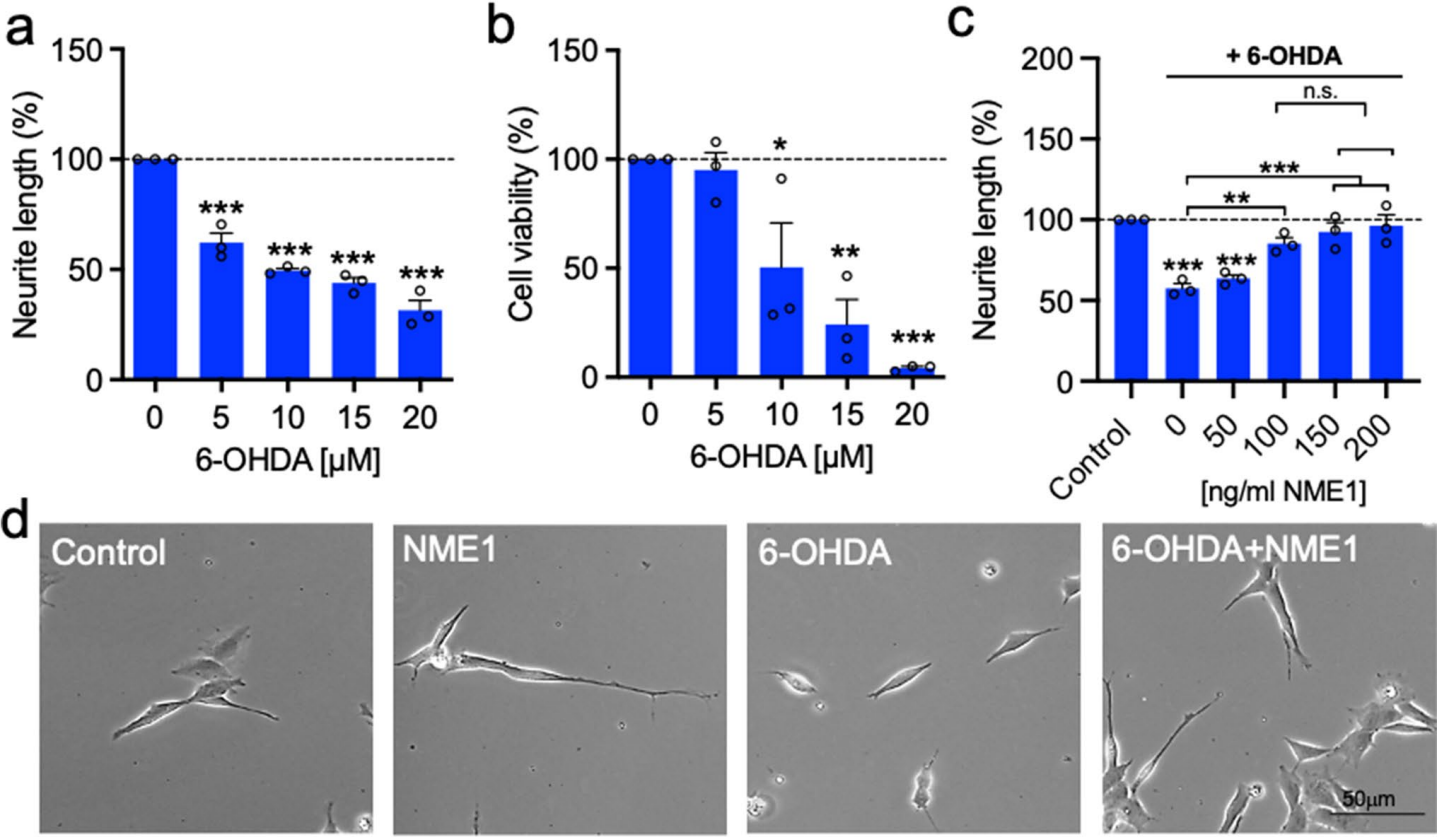
Recombinant NME1 protects against 6-OHDA-induced impairments in neurite growth in SH-SY5Y cells. **a**, **b** Graphs showing **a** neurite length and **b** cell viability expressed as percentages of the control, in SH-SY5Y cells treated with increasing concentrations of 6-OHDA. **c** Graph of neurite length expressed as percentage of the control, in SH-SY5Y cells treated with increasing concentrations of NME1 in the presence of 5 ﻿mM 6-OHDA. **d** Representative phase contrast images of SH-SY5Y cells treated with or without 5 mM 6-OHDA and with or without 100 ng/mL NME1 for 48 h. Data are mean ± SEM from three independent experiments (*p < 0.05, **p < 0.01, ***p < 0.001 versus Control (no treatment) or as indicated; **a**, **b** one-way ANOVA with *post-hoc* Dunnett’s test; **c** one-way ANOVA with *post-hoc* Tukey’s test)

To test the hypothesis that NME1 is neuroprotective, we firstly examined whether treatment with recombinant NME1 could protect against 6-OHDA-induced reductions in neurite growth. We established a model of 6-OHDA-induced neurite injury in SH-SY5Y cells, whereby neurite length was analysed as a readout at a single cell level. To do this, SH-SY5Y cells were treated with 0–20 μM 6-OHDA daily for 48 h and neurite length was measured. This analysis showed that there was a significant decrease in neurite length at concentrations of 6-OHDA ≥ 5 μM, after 48 h (Fig. 1a). There was a significant effect of 6-OHDA on cell viability as measured using MTT assays, with *post-hoc* testing revealing a significant decrease in cell viability after 72 h at concentrations ≥ 10 μM (Fig. 1b). Therefore, to avoid any potential confounding effect of differences in cell viability, treatment with 5 μM 6-OHDA for 48 h was chosen for subsequent experiments. Increasing concentrations (0–200 ng/mL) of recombinant human NME1 were added for 48 h to cells that had been treated with 5 μM 6-OHDA. Treatment with NME1 prevented 6-OHDA-induced reductions in neurite growth (Fig. 1c).

### Recombinant NME1 Promotes Neurite Growth in SH-SY5Y Cells and in Cultured Dopaminergic Neurons Treated with 6-OHDA

**Fig. 2:**
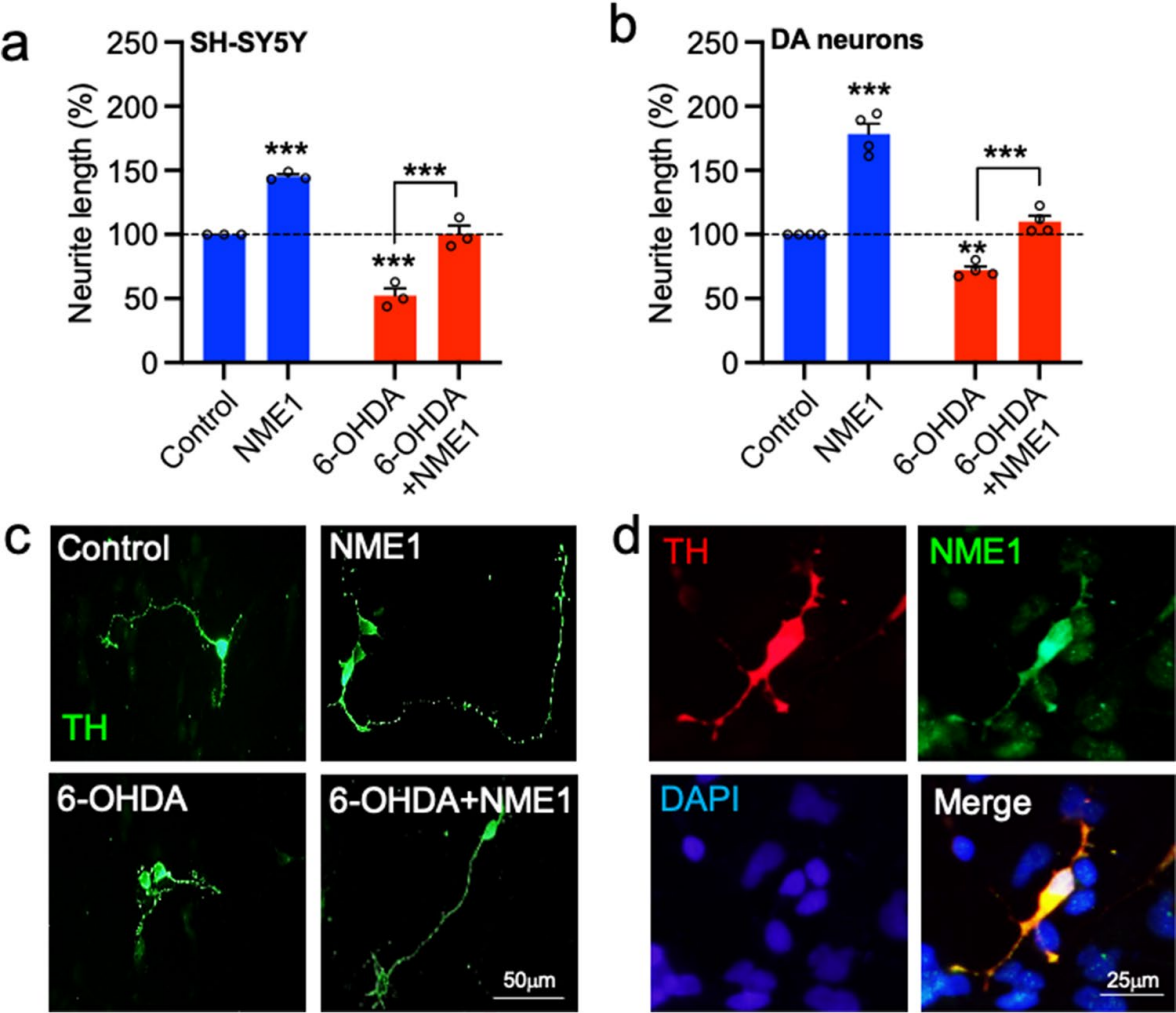
NME1 promotes neurite growth in cultured dopaminergic neurons treated with 6-OHDA. **a**, **b** Graphs of neurite length as percentage of control in **a** SH-SY5Y cells and **b** tyrosine hydroxylase (TH)-positive dopaminergic (DA) neurons in primary cultures of E14 rat VM, that were cultured with or without 5 mM 6-OHDA and with or without 100 ng/mL NME1 for 48 h.. Data are mean ± SEM from three-four independent experiments (**p < 0.01, ***p < 0.001 versus Control (no treatment) or as indicated; two-way ANOVA with *post-hoc* Tukey’s test). **c** Representative photomicrographs of TH-positive DA neurons in each group of **b**. **d** Representative photomicrographs of  of E14 rat VM cultures immunocytochemically stained  for TH (red), NME1 (green) and DAPI (blue). Scale bars as indicated

We next examined the effects of NME treatment on 6-OHDA-induced reductions in neurite growth in SH-SY5Y cells and in primary cultures of dopaminergic neurons. SH-SY5Y cells were cultured with or without 5 μM 6-OHDA and/or 100 ng/mL NME1 for 48 h, and neurite length was analysed as a readout of potential protective effects at a single cell level. A two-way ANOVA revealed significant effects of 6-OHDA (F_(1,8)_ = 107.7, *p* < 0.0001) and of NME1 (F_(1,8)_ = 109.2, *p* < 0.0001) on neurite length, with no significant interaction (F_(1, 8)_ = 0.098, *p* = 0.7618). *Post-hoc* testing showed that recombinant NME1 induced a significant increase in neurite length (*p* = 0.0004) (Fig. 2a, b). In contrast, 6-OHDA treatment resulted in a significant reduction in neurite length (*p* = 0.0003) that was not seen in cultures that were co-treated with NME1 (Fig. 2a, b). Next, primary cultures of E14 rat VM were treated with or without 5 μM 6-OHDA and/or 100 ng/mL NME1 for 48 h and the neurite lengths of individual TH-positive (TH^+^) neurons were analysed. A two-way ANOVA revealed significant effects of both 6-OHDA (F_(1,12)_ = 100.9, *p* < 0.0001) and NME1 (F_(1,12)_ = 147.3, *p* < 0.0001) on neurite length, with a significant drug × toxin interaction (F_(1, 12)_ = 17.93, *p* = 0.0012). *Post-hoc *testing showed that recombinant NME1 led to a significant increase in dopaminergic neurite length (*p* < 0.0001) (Fig. 2c, d). In agreement with the results in SH-SY5Y cells, 6-OHDA led to a significant reduction in neurite length (*p* = 0.0069), that was not seen in cultures co-treated with NME1, when compared to the controls (Fig. 2c, d). Finally, we sought to confirm that dopaminergic neurons in these cultures normally express NME1. Immunocytochemistry revealed that TH+ neurons in primary cultures of E14 rat VM express NME1 in both soma and neurites (Fig. 2e). Collectively, these data show that recombinant NME1 can promote neurite growth in SH-SY5Y cells and in dopaminergic neurons treated with 6-OHDA.

### Recombinant NME1 Promotes Neurite Growth in SH-SY5Y Cells Overexpressing Wild-Type α-Synuclein

**Fig. 3:**
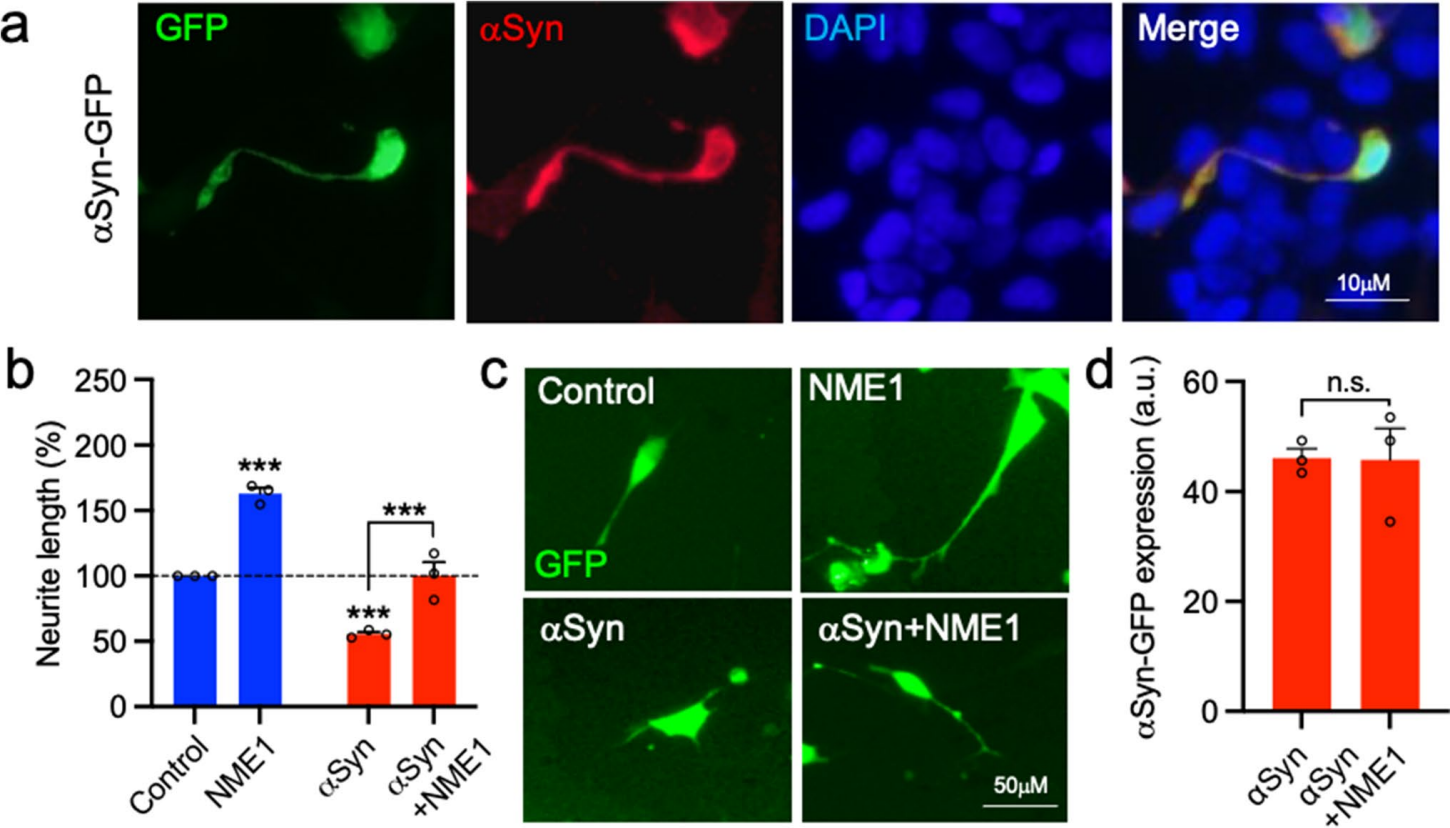
NME1 promotes neurite growth in SH-SY5Y cells overexpressing wild-type a-synuclein. **a** Representative photomicrographs showing ﻿a-synuclein (aSyn) staining in SH-SY5Y cells at 48 h post-transfection with a plasmid expressing GFP-tagged wild-type aSyn (aSyn-GFP). **b** Graph of neurite length as percentage of control and **c** representative photomicrographs of SH-SY5Y cells transfected with plasmids expressing GFP or aSyn-GFP and cultured with or without 100 ng/mL NME1 for 48 h. **d** Graph showing  aSyn-GFP expression in arbitrary units (a.u.) in both groups transfected with the aSyn-GFP plasmid. Data are mean ± SEM from three independent experiments (*n* = 3) (***p < 0.001 versus Control (no treatment) or as indicated; **b** two-way ANOVA with *post-hoc* Tukey’s test), **d** Student’s *t*-test

We next examined the effects of recombinant NME1 in cellular models of PD generated by the overexpression of wild-type (WT) α-synuclein. SH-SY5Y cells were transfected with either a control-GFP plasmid (Control) or a plasmid expressing GFP-tagged WT α-synuclein (α-syn), and then were cultured with or without 100 ng/mL NME1 for 48 h. Immunocytochemistry revealed that overexpression of α-synuclein led to strong intracellular expression of α-synuclein in transfected cells (Fig. 3a). A two-way ANOVA revealed significant effects of both α-synuclein (F_(1, 8)_ = 91.14, *p* < 0.0001) and NME1 (F_(1,8)_ = 92.08, *p* < 0.0001) on neurite length, with no significant interaction (F_(1,8)_ = 2.648, *p* = 0.1423). *Post-hoc* testing showed that recombinant NME1 significantly increased neurite length (*p* = 0.0002) (Fig. 3b, c). In contrast, α-synuclein significantly reduced neurite length (*p* = 0.0023), an effect that was not seen when cells were co-treated with NME1 (Fig. 3b, c). Furthermore, we report that the expression of GFP-α-syn was uniform in the cells that had been treated with or without NME1 (Fig. 3d).

### Recombinant NME1 Promotes Neurite Growth in Cultured Dopaminergic Neurons Overexpressing Human Wild-Type α-Synuclein

**Fig. 4:**
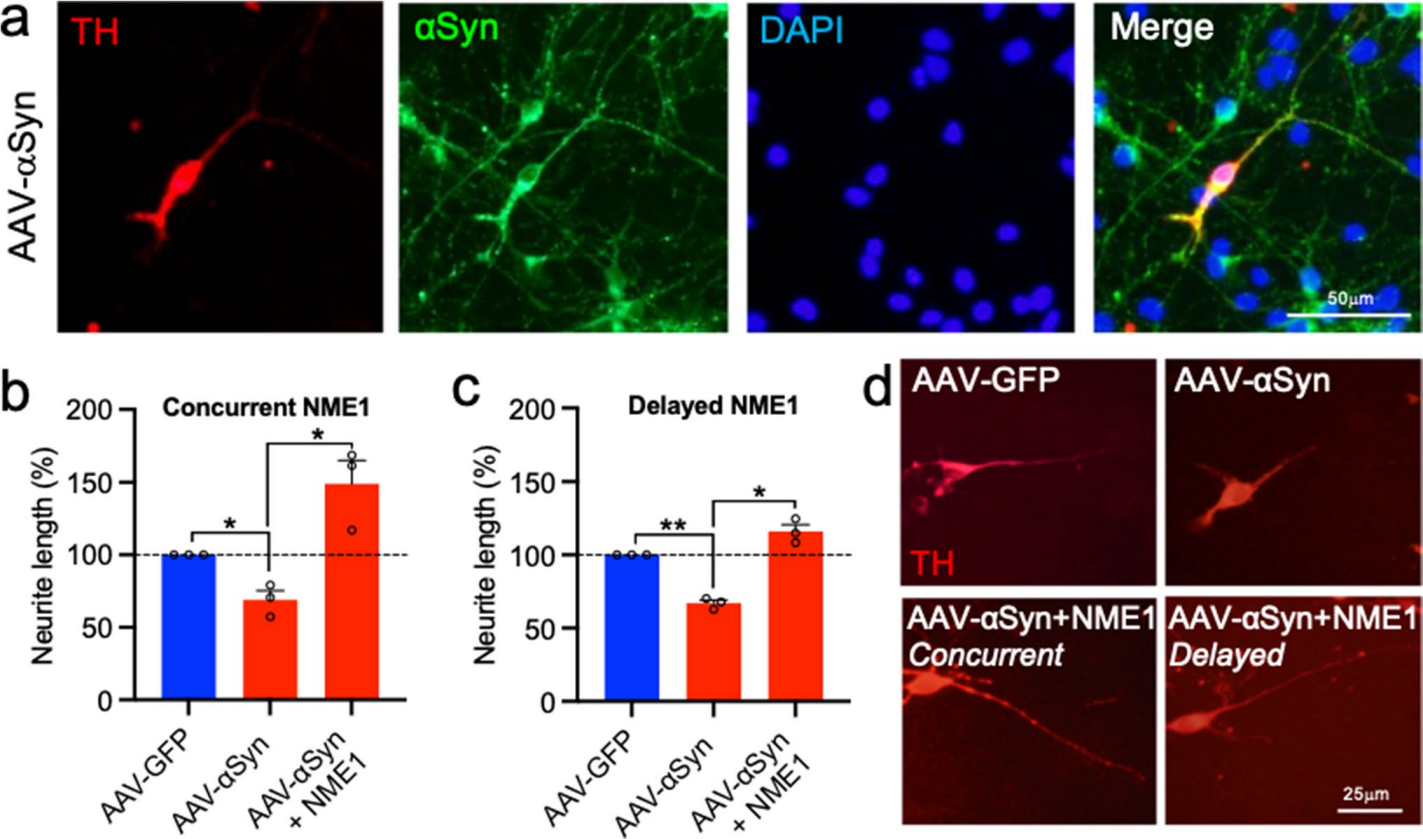
NME1 promotes neurite growth in cultured dopaminergic neurons overexpressing human wild-type a-synuclein. **a** Representative photomicrographs showing human a-synuclein (aSyn) staining (green) staining in TH-positive (red) dopaminergic neurons in primary cultures of E14 rat VM at 5 days post-transduction with an AAV-aSyn vector. Cells were counterstained with DAPI (blue). **b**, **c** Graphs of neurite length as percentage of the control (AAV-GFP) and **d** representative photomicrographs of TH-positive neurons transduced with an AAV-GFP or AAV-aSyn vector and cultured without or with 100 ng/mL NME1 which was added either **b** daily at the time of transduction for 10 days (concurrent), or **c** 5 days post-transduction for an additional 5 days (delayed). Data are mean ± SEM from three independent experiments (*n* = 3) (*p < 0.05, **p < 0.01 versus Control (AAV-GFP with no treatment) or as indicated; one-way ANOVA with *post-hoc* Fisher’s LSD test)

 beneficial effects of recombinant NME1 in SY-SY5Y cells, we next sought to confirm these findings in cultured dopaminergic neurons. To do this, primary cultures of E14 rat VM were transduced with an AAV-α-synuclein (AAV-αSyn) vector, which resulted in widespread expression of α-synuclein in TH-positive neurons in these cultures (Fig. 4a). To confirm the effects of AAV-αSyn, control cultures were transduced with an AAV-GFP vector. We next performed two experiments using two different treatment regimens of NME1. In experiment 1, primary cultures of E14 rat VM were transduced with either AAV-GFP, AAV-αSyn or AAV-αSyn in combination with 100 ng/mL NME1 daily for 10 days, before the neurite length of individual TH-positive neurons was analysed (concurrent treatment). Analysis showed that AAV-αSyn resulted in a significant reduction in dopaminergic neurite length (*p* = 0.0397); this was not seen when these cells were treated with recombinant NME1, which were significantly longer than those with AAV-αSyn alone (Fig. 4b, c). In experiment 2, primary cultures of E14 rat VM were transduced with AAV-GFP, AAV-αSyn or AAV-αSyn in combination with 100 ng/mL NME1 daily from 5 days post-transduction, for an additional for 5 days (delayed treatment). Analysis of the neurite length of individual TH-positive neurons revealed that AAV-αSyn resulted in a significant reduction in dopaminergic neurite length (*p* = 0.0043) that was not seen when these cells were treated with recombinant NME1 (Fig. 4d, e). Collectively, these data show that recombinant NME1 prevents α-synuclein-induced reductions in dopaminergic neurite length.

### Recombinant NME1 Promotes Neurite Growth in Stable G2019S LRRK2 SH-SY5Y Cells

**Fig. 5:**
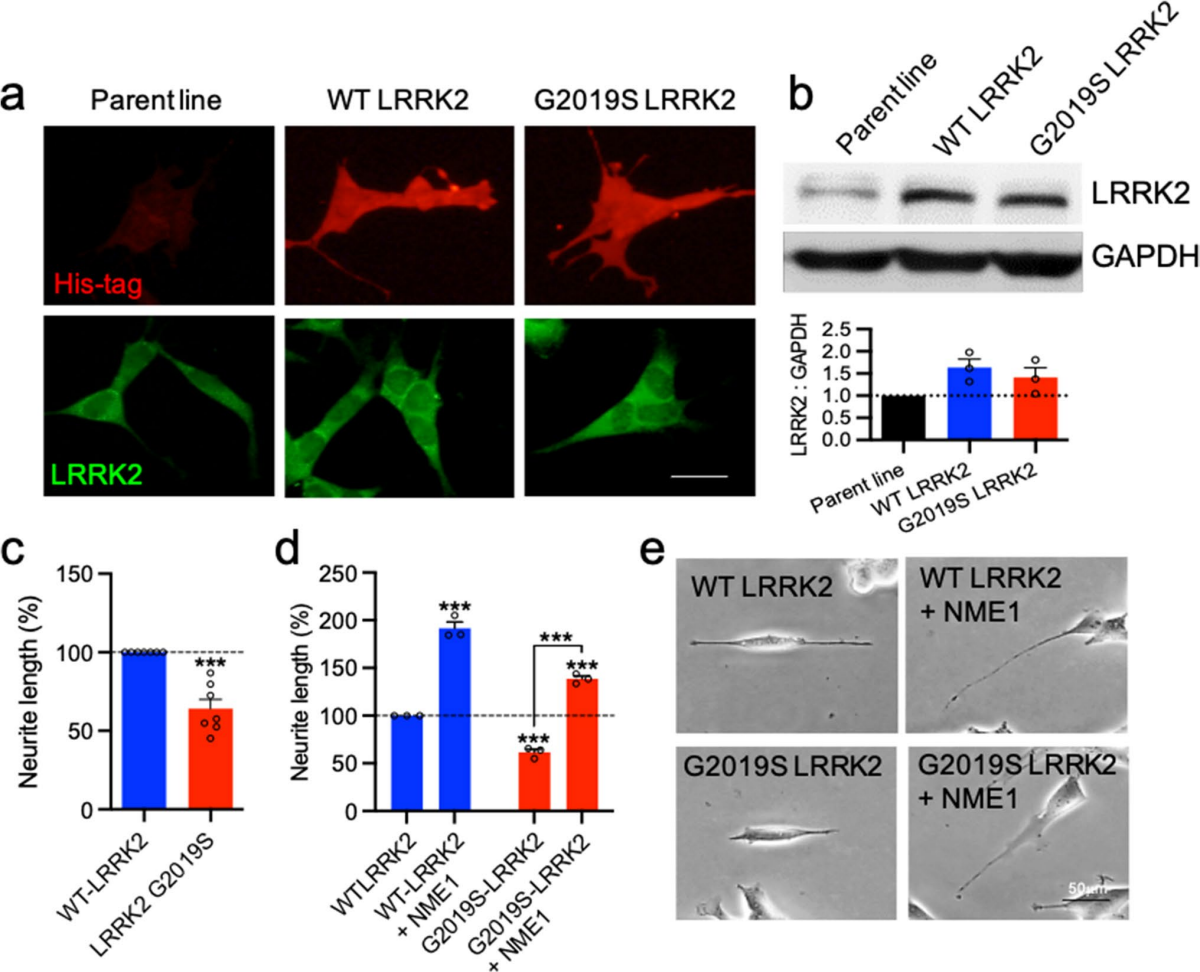
NME1 promotes neurite growth in stable G2019S LRRK2 SH-SY5Y cells. **a** Representative photomicrographs showing His-tag staining (red) and LRRK2 staining (green) and **b** western blotting and graph showing the expression of LRRK2 relative to GAPDH expression in parent, wild-type (WT) LRRK2 and G2019S LRRK2 stable SH-SY5Y cell lines at 72 h. **c**, **d** Graphs of neurite length in each cell line, as percentages of that in WT LRRK2 cells. **e** Representative photomicrographs of WT LRRK2 and G2019S LRRK2 SH-SY5Y cells cultured with or without 100 ng/mL NME1 for 48 h. Data are mean ± SEM from three to seven independent experiments (***p < 0.001 versus the relevant Control or as indicated. **b** One-way ANOVA, **c** Student’s *t*-test, **d** two-way ANOVA with *post-hoc* Tukey’s test)

Next, we examined the effects of recombinant NME1 in WT LRRK2 and G2019S LRRK2 stably-transfected SH-SY5Y cell lines (Fig. 5a). Western blotting for LRRK2 protein expression confirmed comparable levels of LRRK2 expression in SH-SY5Y cells stably expressing either WT LRRK2 or G2019S LRRK2 (Fig. 5b). We observed a significant reduction in neurite growth in cells stably expressing G2019S LRRK2, compared to cells stably expressing WT LRRK2 (Fig. 5c). A two-way ANOVA revealed significant effects of LRRK2 (F_(1,8)_ = 127.2, *p* < 0.0001) and of NME1 (F_(1,8)_ = 432.7, *p* < 0.0001) on neurite length, with no significant interaction (F_(1, 8)_ = 3.133, *p* = 0.1147). *Post-hoc* testing showed that NME1 significantly increased neurite length (*p* < 0.0001) (Fig. 5d, e). G2019S-LRRK2 significantly reduced neurite length (*p* = 0.0007), an effect that was not seen in G2019S-LRRK2 cultures co-treated with NME1 (Fig. 5d, e). These data show that recombinant NME1 can promote neurite growth in cells overexpressing α-synuclein or G2019S-LRRK2.

### ROR2 and RORα are Required for the Neurite Growth-Promoting Effects of NME1

**Fig. 6:**
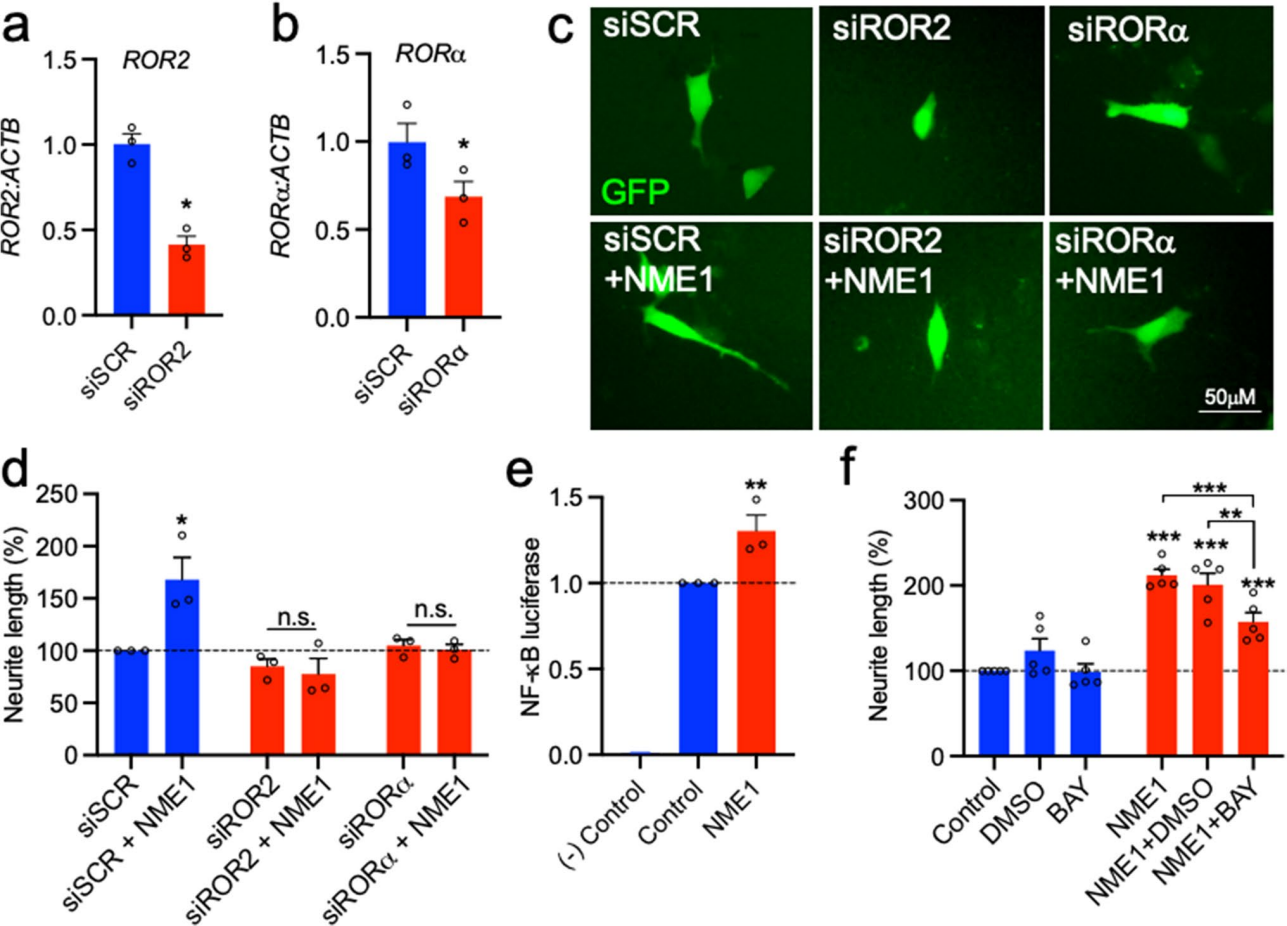
ROR2 and RORa are required for the neurite growth-promoting effects of NME1. **a**, **b** Real time PCR showing the expression of **a** *ROR2* and **b** *RORa* normalised to the expression of *ACTB* in SH-SY5Y cells transfected with a scrambled siRNA (siSCR) or siRNAs targeting *ROR2* (siROR2) or *RORa* (siRORa) for 48 h. **c** Representative phase photomicrographs of SH-SY5Y cells transfected with a scrambled siRNA (siSCR), siROR2 or siRORa and cultured with or without 100 ng/mL NME1 for 48 h. **d** Graphs of neurite length as percentage of control of SH-SY5Y cells transfected with a scrambled siRNA (siSCR), siROR2 or siRORa and cultured with or without 100 ng/mL NME1 for 48 h. **e** Graph of relative luciferase units (R.L.U.) in SH-SY5Y cells transfected with 100 ng of an NF-kB firefly luciferase reporter and 1 ng of a renilla reporter plasmid. Untransfected cells were used as a negative control [(−) Control]. **f** Graphs of neurite length as percentage of control of SH-SY5Y cells treated with or without DMSO or BAY inhibitor. Where indicated cells were treated with 100 ng/mL for 48 h. Data are mean ± SEM from three-five independent experiments (*p < 0.05, **p < 0.01 versus relevant Control; n.s. = not significant; **a**, **b** Paired Student’s *t*-test; **d**, **f** Two-way ANOVA with *post-hoc* Tukey’s test; **e** One-way ANOVA with *post-hoc* Fisher’s LSD test

We next sought to explore the mechanisms involved in NME1-promoted neurite growth. Previous studies had shown that NME1 physically interacts with ROR receptors [33] and that ROR2 is a non-canonical receptor for GDF5 [34], which induces NME1 expression [21]. Therefore, we hypothesised that ROR2 and/or RORα may be required for the neurotrophic effects of NME1. To test this hypothesis, SH-SY5Y cells were transfected with a scrambled siRNA (siSCR), or with siRNAs targeting ROR2 (siROR2) or RORα (siRORα), together with a GFP-expressing plasmid to identify transfected cells, then cultured with or without 100 ng/mL NME1 for 48 h, before neurite length was measured. To verify that the siRNA were target-specific, we transfected SH-SY5Y cells with siRNA targeting RORα and ROR2, or with scrambled negative control siRNA. RNA for *ROR2* and for *RORα* were both significantly down regulated at 48 h post-transfection (Fig. 6a, b). A two-way ANOVA revealed a significant interaction effect of siRNA × NME1 (F_(2, 12)_ = 7.035, *p* = 0.0095). *Post-hoc* testing showed that NME1 significantly increased neurite length (*p* = 0.0113) in cells transfected with siSCR (Fig. 6c, d). In contrast, NME1 did not promote neurite growth in siROR2- or siRORα-transfected cells (Fig. 6c, d). Collectively, these data show that ROR2 and RORα are required for the neurite growth-promoting effects of NME1.

### NME1 Stimulated an NF-κB Transcriptional Response and Pharmacological Inhibition of NF-κB Partially Prevented NME1-Mediated Neurite Growth in SH-SY5Y Cells

We next sought to understand the cellular pathway through which NME1 promotes neurite growth. NME1 has previously been demonstrated to upregulate the NF-κB pathway in human embryonic kidney (HEK) 293 T cell lines [35, 36]. It is known that NF-κB can promote or inhibit neurite growth, depending on the cellular context [37]. Therefore we examined whether the NF-κB transcriptional response was required for the neurite growth-promoting effects of NME1 in our cells. To do this, SH-SY5Y cells were transfected with an NF-κB firefly luciferase reporter, along with a control renilla luciferase reporter plasmid, and cultured with or without 100 ng/ml of NME1. A one-way ANOVA revealed that there was a significant effect of NME1 treatment on NF-κB-dependent transcription (F_(2, 6)_ = 162.9, *p* < 0.0001), with a *post-hoc* analysis revealing that NME1 treatment resulted in a significant increase in the NF-κB luciferase reporter activity in comparison to the control (*p* = 0.0067) (Fig. 6e). Since NME1 treatment upregulated NF-κB, we examined whether inhibition of NF-κB would prevent the effects of NME1 on neurite growth. To do this, SH-SY5Y cells were treated with or without NME1, and with the NF-κB inhibitor BAY, and neurite length was examined at 48 h. A two-way ANOVA revealed a significant effect of NME1 on neurite growth (F_(1, 16)_ = 117.6, *p* < 0.0001). A subsequent *post-hoc* analysis revealed that while BAY did not impede basal levels of neurite growth, it partially reduced NME1-mediated neurite growth (Fig. 6f). Taken together, these findings suggest that NME1-mediated NF-κB dependent transcription is at least partially required for the effects of NME1 on neurite growth in SH-SY5Y cells.

### Bioinformatics and Bioenergetic State Analysis Implicates NME1 as a Modulator of Mitochondrial Function

**Fig. 7:**
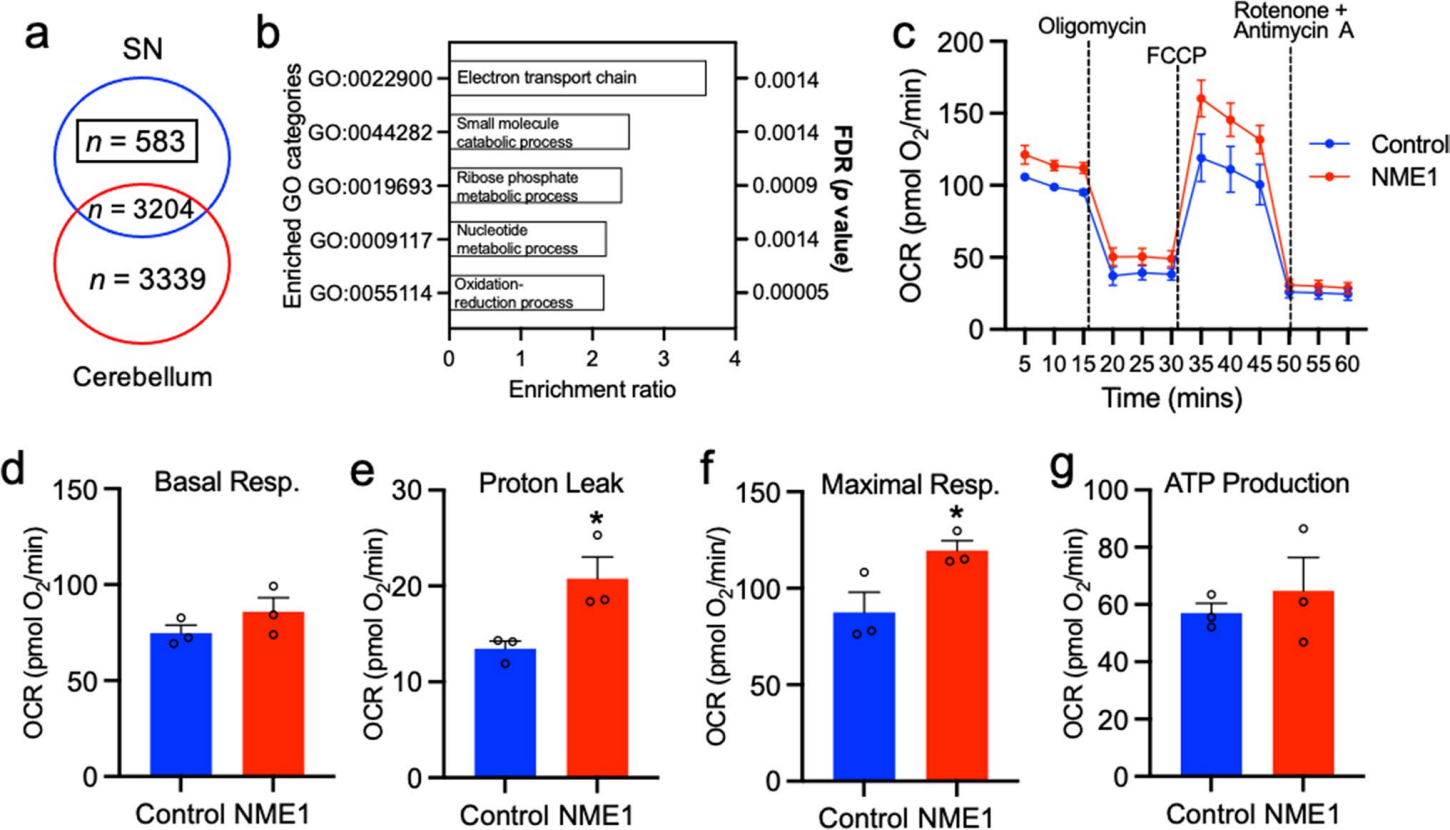
Gene co-expression analysis suggests a role for NME1 in mitochondrial function, and NME1 alters the oxygen consumption rate in SH-SY5Y cells. **a** Venn diagram showing the numbers of NME1 co-expressed genes from gene co-expression analysis of *NME1*in the human substantia nigra (SN) and cerebellum. Raw data were derived from data set GSE60863 and analyzed using the R2 microarray platform. **b** Graph showing the significantly enriched GO categories from the *n* = 583 genes correlated with NME1 in the SN. Analysis was performed using www.geneontology.org. **c** Graph of data from Seahorse assays showing the mitochondrial oxygen consumption rate (OCR) of SH-SY5Y cells treated with 100 ng/mL NME1. **d**–**g** Graphs showing **d** basal respiration, **e** proton leak, **f** maximal respiration and **g** ATP production. Data are mean ± SEM, *n* = 3 independent experiments. *p < 0.05, **p < 0.01, and ***p < 0.001 versus untreated; **d**–**g** Student’s *t*-test

We next sought to gain insight into the cellular processes that may be regulated by NME1. Gene co-expression network analysis is a bioinformatics approach that can be used to associate genes of unknown function with specific biological processes, as genes that have a functional relationship display a correlated pattern of expression [38]. We used a large transcriptome data set from brain regions of 134 healthy human controls (GSE:60863, [39]) and performed a pairwise correlation analysis between *NME1* and every other gene in the SN (*n* = 101) and the cerebellar cortex (*n* = 130) (as a control). This analysis identified 583 genes that had a significantly correlated pattern of expression with *NME1,* after a Bonferroni-corrected multiple testing, that were unique to the SN (Fig. 7a). We then performed a gene ontology (GO) enrichment analysis which revealed that the top GO category was ‘electron transport chain’ (GO:0022900) (Fig. 7b). This suggested that NME1 may modulate mitochondrial function.

To investigate the hypothesis that NME1 can modulate mitochondrial function, we performed an analysis of cellular bioenergetic state by measuring the oxygen consumption rate in SH-SY5Y cells treated with 100 ng/mL NME1, using the Seahorse XF 96 Extracellular Flux Analyser. NME1-treated cells showed enhancements in respiration function during the experiment, compared to the controls (Fig. 7c). Specifically, the levels of basal respiration were unaffected by NME1 (Fig. 7c, d). Addition of oligomycin, an inhibitor of mitochondrial ATP-synthase, caused a decrease in ATP production and there was a significant increase in mitochondrial ATP-synthase in NME1-treated cells (Fig. 7c, e). Subsequent addition of FCCP caused uncoupling of mitochondrial oxidative phosphorylation (OX-PHOS) to induce maximal respiration. NME1 treatment led to a significant increase in maximal respiration capacity, which is indicative of uncoupling (Fig. 7c, f). Finally, a combination of rotenone and antimycin A was added to inhibit complex I of the mitochondrial respiration chain and there was no notable effect of NME1 on the spare capacity (Fig. 7c, g). Collectively, these data show that NME1 modulates mitochondrial function in SY-SY5Y cells.

## Discussion

Neurite degeneration of dopaminergic neurons is now recognised to be a core part of the cellular pathology of PD [5, 23]. We have recently shown that recombinant NME1 treatment can increase neurite and axonal growth in SH-SY5Y cells and dopaminergic neurons [21]. In that study, we described the beneficial effects of recombinant NME1 on the promotion of neurite growth in cell models of PD. We first showed that treatment with recombinant NME1 could protect against 6-OHDA-induced neurite degeneration in SH-SY5Y cells. This is consistent with several previous studies, which showed that treatment with GDF5 exerts protective effects against 6-OHDA-induced dopaminergic neuron degeneration in vitro [40, 41] and in vivo [42–44].

However, while neurotoxins such as 6-OHDA are useful for modelling dopaminergic axonal degeneration, PD is characterised by the cellular accumulation of α-synuclein. Therefore, in this study we examined the effects of NME1 treatment in cellular models of PD that involved overexpression of α-synuclein. We found that overexpression of wild-type human α-synuclein in human SH-SY5Y cells, or in cultured rat dopaminergic neurons, reduced neurite growth. This is in agreement with previous studies showing that α-synuclein reduces neurite growth in SH-SY5Y cells [45, 46], primary dopaminergic neurons [47], and induced pluripotent stem cell (iPSC)-derived dopaminergic neurons from patients carrying the A53T mutation in α-synuclein [48]. Importantly, we found that treatment with recombinant NME1 prevented the detrimental effects of α-synuclein on neurite growth. We also demonstrated that delayed NME1 treatment was effective in cultured dopaminergic neurons which had an already established α-synuclein burden, which is an important proof-of-concept finding, as this model better reflects the neuropathological features of the clinical scenario. Moreover, both GDF5 [49] and BMP5/BMP7 [50] have recently been shown to protect against α-synuclein-induced dopaminergic degeneration in rat and mouse models of PD, and GDF5 is known to induce NME1 expression in vitro and in vivo [21]. These findings suggest that the beneficial effects of GDF5, and perhaps of other BMP ligands, in vivo may require up-regulation of NME1. In future work, it will be important to determine the precise contribution of NME1 to the neurotrophic effects of these ligands in vivo, and to determine whether NME1 can protect against α-synuclein-induced degeneration in vivo*.*

While the investigation of neurotrophic agents in models of α-synucleinopathy is important, it is unclear if these same agents would be beneficial in other forms of PD. The G2019S mutation in LRRK2 is the most common cause of familial PD, and LRRK2 G2019S has been reported to impair neurite growth [51]. Therefore, we also examined in this study whether NME1 could protect against LRRK2 G2019S-induced degeneration. We generated SH-SY5Y-derived cell lines that stably overexpressed LRRK2 wild-type and G2019S. In agreement with previous studies [51], we found that LRRK2 G2019S impaired neurite growth, an effect that was not seen when these cells were treated with NME1. This effect of NME, the downstream mediator of GDF5 signalling, to protect against reductions in neurite growth induced by the LRRK2 G2019S mutation, is not common to all neurotrophic factors. For example, the related neurotrophic factor, glial cell line-derived neurotrophic factor (GDNF), has previously been shown to be incapable of restoring neurite growth in cells carrying LRRK2 G2019S mutations. Specifically, GDNF-containing differentiation protocols applied to iPSCs from PD patients carrying LRRK2 G2019S mutation resulted in impairments in early neurite branching and growth [52].

In this study, we also showed that the neurite growth-promoting effects of NME1 are dependent upon ROR receptors. NME1 has been reported to interact with RORa [33], and RORa is thought to be a candidate gene for sporadic PD [53]. Additionally, as retinoic acid exerts its differentiation-stimulating effects through ROR2 and RORa in cultured mouse hippocampal neurons and neuroblastoma cell lines [54–56], we examined the roles of both RORa and ROR2 receptors in NME1-promoted neurite growth. We found that NME1 required both RORa and ROR2 to promote neurite growth in SH-SY5Y cells. This is an important finding in the wider context of neurotrophic factor therapy for PD. To date, clinical trials of GDNF therapy in PD have been unsuccessful [57, 58]. Moreover, GDNF failed to protect against α-synuclein-induced degeneration in a rat model of PD [59]. This is thought to be due to an α-synuclein-induced down-regulation of expression of the GDNF receptor, Ret [60]. Because of this, it has been proposed that Ret-independent neurotrophic factors may be more effective as therapeutic agents than GDNF and neurturin, which are both dependent on signalling through Ret for their dopaminergic neurotrophic effects. Our finding that NME1 exerts its effects through RORa and ROR2, and not through Ret, is consistent with our data showing that NME1 can protect against α-synuclein-induced degeneration in vitro.

This study demonstrated that NME1 activated NF-κB-dependent gene transcription, and that pharmacological inhibition of NF-κB partially prevented the effects of NME1 on neurite growth. This is consistent with previous reports showing that NME1 upregulates the NF-κB pathway and the expression of NF-κB targets in HEK293T cells [35, 36]. Interestingly the NF-κB pathway has been reported to be negatively regulated in *SNCA* models of PD and in 1-methyl-4-phenylpyridinium (MPP+) in vivo models of PD [61]. Furthermore, NF-κB restoration has also been considered in therapeutic approaches for PD [62]. In this context, it will be important to fully decipher the intracellular mechanisms of NME1-induced NF-κB activation and to explore the potential of this approach for PD treatment.

We also explored the cellular processes that may be influenced by NME1 using gene co-expression network analysis. This is a bioinformatics approach that is used to associate genes of unknown function with specific biological processes, as genes that have a functional relationship display a correlated pattern of expression [38]. To do this, we generated a list of genes that are uniquely co-expressed with NME1 in the human SN; this list was enriched in genes associated with the electron transport chain and with oxidation–reduction processes. This made a strong case for the assessment of the impact of NME1 on cellular respiration. In agreement with this, our findings from the Seahorse assays demonstrated increases in proton leak and in maximal respiration in NME1-treated SH-SY5Y cells. These data are important as it has been shown that increasing or restoring maximal respiration is neuroprotective in SH-SY5Y cells and in cultured cortical neurons in Aβ models of neurodegeneration [63]. Increments in proton leak are also known to reduce reactive oxygen species through a feedback mechanism involving the recruitment of superoxide dismutase, which provides cytoprotection [64]. In the context of PD, this is important as human neuroepithelial stem cells derived from PD patients with the LRRK2 G2019S mutation have lowered levels of basal and maximal respiration [65]. Furthermore, dopaminergic neurons derived from human iPSCs carrying the SNCA A53T mutation showed decreases in basal respiration, ATP production, maximal respiration capacity and spare respiratory capacity [66]. The fact that NME1 increases mitochondrial respiration, together with the finding that NME1 protects against α-synuclein and G2019S LRRK2-induced degeneration, is an important proof-of-principle that NME1 may be a promising therapeutic agent for neuroprotection in PD.

We have demonstrated that NME1 treatment promotes neurite growth in several cellular models of PD, and that NME1 can restore mitochondrial respiration and cellular pathways which are known to be impaired in PD. It is noteworthy that NME1 copy number reductions at the 3-prime end of NME1 gene have been reported in dementia with Lewy body (DLB) and that in PD, NME1 transcripts have been found to be reduced in comparison to controls (Kun-Rodrigues et al., 2019) [21]. This lends support to the proposal that NME1 is a potential candidate for therapeutic approaches to neurodegenerative disease.

Whilst the use of NME1 is still at an early stage of study for application in PD therapy, we propose to evaluate the application of NME1 in further studies, using various modes of delivery. These include, but are not limited to, stereotactic injection of recombinant NME1 and AAV-mediated gene delivery of NME1 into the adult rat SN, as well as investigation of NME1 delivery through blood or intrathecal routes [67–69]. A key question for future research will be whether NME1 can act in a retrograde fashion if administered to the striatum or whether it will require administration to the SN. Such studies of NME1 and its roles in alleviating neurodegeneration *in vivo* will be required to rationalise the continued investigation of NME1 as a potential therapeutic approach for neuroprotection in PD.

## Data Availability

All data generated during this study are included in this article or are available on reasonable request from the corresponding authors.

## Abbreviations

AAV: Adeno-Associated Viral Vector
CNS: Central Nervous System
DMEM: Dulbecco’s Modified Eagle’s Medium
E: Embryonic Day
FBS: Foetal Bovine Serum
GDF5: Growth Differentiation Factor 5
GDNF: Glial Cell Line-Derived Neurotrophic Factor
GFP: Green Fluorescent Protein
GO: Gene Ontology
HBSS: Hanks Balanced Salt Solution
6-OHDA: 6-Hydroxydopamine
LRRK2: Leucine-Rich Repeat Kinase 2
MPP+: 1-Methyl-4-Phenylpyridinium
MOI: Multiplicity of Infection
NME1: Nucleoside Diphosphate Kinase A
OCR: Oxygen Consumption Rate (OCR)
PBS-Tx: PBS with Triton-X100
PD: Parkinson’s Disease
Si: SiRNA
SN: Substantia Nigra
SNCA: α-Synuclein Gene
αSyn: α-Synuclein
TGF: Transforming Growth Factor
TH: Tyrosine Hydroxylase
VM: Ventral Mesencephalon
WT: Wild-Type
